# The effect of meal frequency in a reduced-energy regimen on the gastrointestinal and appetite hormones in patients with type 2 diabetes: A randomised crossover study

**DOI:** 10.1371/journal.pone.0174820

**Published:** 2017-04-03

**Authors:** Lenka Belinova, Hana Kahleova, Hana Malinska, Ondrej Topolcan, Jindra Windrichova, Olena Oliyarnyk, Ludmila Kazdova, Martin Hill, Terezie Pelikanova

**Affiliations:** 1 Department of Diabetology, Institute for Clinical and Experimental Medicine, Prague, Czech Republic; 2 First Faculty of Medicine, Charles University, Prague, Czech Republic; 3 Laboratory of Immunoanalysis, University Hospital in Pilsen, Pilsen, Czech Republic; 4 Department of Steroid Hormones and Proteohormones, Institute of Endocrinology, Prague, Czech Republic; Garvan Institute of Medical Research, AUSTRALIA

## Abstract

**Background:**

Appetite and gastrointestinal hormones (GIHs) participate in energy homeostasis, feeding behavior and regulation of body weight. We demonstrated previously the superior effect of a hypocaloric diet regimen with lower meal frequency (B2) on body weight, hepatic fat content, insulin sensitivity and feelings of hunger compared to the same diet divided into six smaller meals a day (A6). Studies with isoenergetic diet regimens indicate that lower meal frequency should also have an effect on fasting and postprandial responses of GIHs. The aim of this secondary analysis was to explore the effect of two hypocaloric diet regimens on fasting levels of appetite and GIHs and on their postprandial responses after a standard meal. It was hypothesized that lower meal frequency in a reduced-energy regimen leading to greater body weight reduction and reduced hunger would be associated with decreased plasma concentrations of GIHs: gastric inhibitory peptide (GIP), glucagon-like peptide-1(GLP-1), peptide YY(PYY), pancreatic polypeptide (PP) and leptin and increased plasma concentration of ghrelin. The postprandial response of satiety hormones (GLP-1, PYY and PP) and postprandial suppression of ghrelin will be improved.

**Methods:**

In a randomized crossover study, 54 patients suffering from type 2 diabetes (T2D) underwent both regimens. The concentrations of GLP-1, GIP, PP, PYY, amylin, leptin and ghrelin were determined using multiplex immunoanalyses.

**Results:**

Fasting leptin and GIP decreased in response to both regimens with no difference between the treatments (*p* = 0.37 and *p* = 0.83, respectively). Fasting ghrelin decreased in A6 and increased in B2 (with difference between regimens *p* = 0.023). Fasting PP increased in B2with no significant difference between regimens (*p* = 0.17). Neither GLP-1 nor PYY did change in either regimen. The decrease in body weight correlated negatively with changes in fasting ghrelin (r = -0.4, *p*<0.043) and the postprandial reduction of ghrelin correlated positively with its fasting level (r = 0.9, p<0.001). The postprandial responses of GIHs and appetite hormones were similar after both diet regimens.

**Conclusions:**

Both hypocaloric diet regimens reduced fasting leptin and GIP and postprandial response of GIP comparably. The postprandial responses of GIHs and appetite hormones were similar after both diet regimens. Eating only breakfast and lunch increased fasting plasma ghrelin more than the same caloric restriction split into six meals. The changes in fasting ghrelin correlated negatively with the decrease in body weight. These results suggest that for type 2 diabetic patients on a hypocaloric diet, eating larger breakfast and lunch may be more efficient than six smaller meals during the day.

## Introduction

Appetite and gastrointestinal peptides play an important role in the regulation of energy intake, appetite and overall energy homeostasis in humans [1, 2]. Their fasting plasma levels could be increased and postprandial responses attenuated by the presence of obesity [3], impaired glucose tolerance [4] and type 2 diabetes mellitus (T2D) [5, 6]. Caloric restriction and body weight reduction are supposed to decrease fasting plasma levels [7, 8] and increase meal responses of gastrointestinal hormones (GIH) with effects on satiety: for instance, glucagon-like peptide-1(GLP-1) and peptide YY (PYY) [9, 10] and reduce postprandial response of gastric inhibitory polypeptide (GIP) [11]. Studies with hypocaloric diet regimens indicate that lower meal frequency should also have an effect on GIH´s postprandial responses: ghrelin and GLP-1 [12]. To the best of our knowledge, the effects of different hypocaloric diet regimens on other GIHs and their postprandial responses have not been published yet.

It has been postulated that increasing eating frequency regimens could help to maintain appetite control, improve glucose metabolism, and therefore reduce body fat storage and body weight [13]. Contrary to this, a very recent meta-analysis has showed that eating more frequently during the day (i.e., “grazing”) may not assist with reducing energy intake or improving weight status [14]. It is suggested that eating more often than three times a day may lead to obesity [15], by increasing food stimuli and appetite [16]. However, there are also studies, which do not support any associations between body weight and eating frequency at all [17, 18].

We have shown previously that in patients with type 2 diabetes less frequent eating(breakfast and lunch consumption)reduced body weight, hepatic fat content, insulin resistance [19] and hunger [20]more than a diet with the same caloric restriction divided into six more frequent meals. The aim of this secondary analysis was to explore the effect of these two hypocaloric diet regimens on the fasting levels of appetite and GIHs and on their postprandial responses after a standard meal. The resulting positive effects following the hypocaloric diet divided into less frequent meals could be due, at least in part, to an alteration in the circulating levels and physiology of certain gastrointestinal and appetite hormones.

Appetite hormones, ghrelin and leptin, are well known to play a prominent role in regulation of energy homeostasis [2]. In obese subjects [21]and patients with T2D, ghrelin secretion is down-regulated and the decline in plasma ghrelin after a meal is blunted [22, 23]. Hypocaloric diet regimen is postulated to improve ghrelin sensitivity in these individuals [24, 25]and improves the postprandial ghrelin response independently on diet composition [26].

Peptide YY (PYY) is co-secreted predominantly from the endocrine L cells in the ileum together with glucagon-like peptide-1 (GLP-1). Pancreatic polypeptide (PP) is secreted from endocrine cells in the pancreas. GLP-1, PYY and PP are supposed to induce satiety and to reduce appetite and energy intake in healthy humans [27–29].

The aim of our study was to compare the effects of two different hypocaloric regimens, six vs. two meals a day (breakfast and lunch; as this regimen allows reasonable fasting time, yet is sustainable in the long term), with the same caloric restriction on fasting levels of appetite and GI hormones and their postprandial responses after a standard meal.

It was hypothesized that lower meal frequency in a reduced-energy regimen leading to greater body weight reduction and reduced hunger would be associated with decreased plasma concentrations of GIHs (GIP, GLP-1, PYY, PP) and leptin and increased plasma concentration of ghrelin. The postprandial response of satiety hormones (GLP-1, PYY and PP) and postprandial suppression of ghrelin will be improved.

## Methods and materials

### Study design and participants

**Fig 1:**
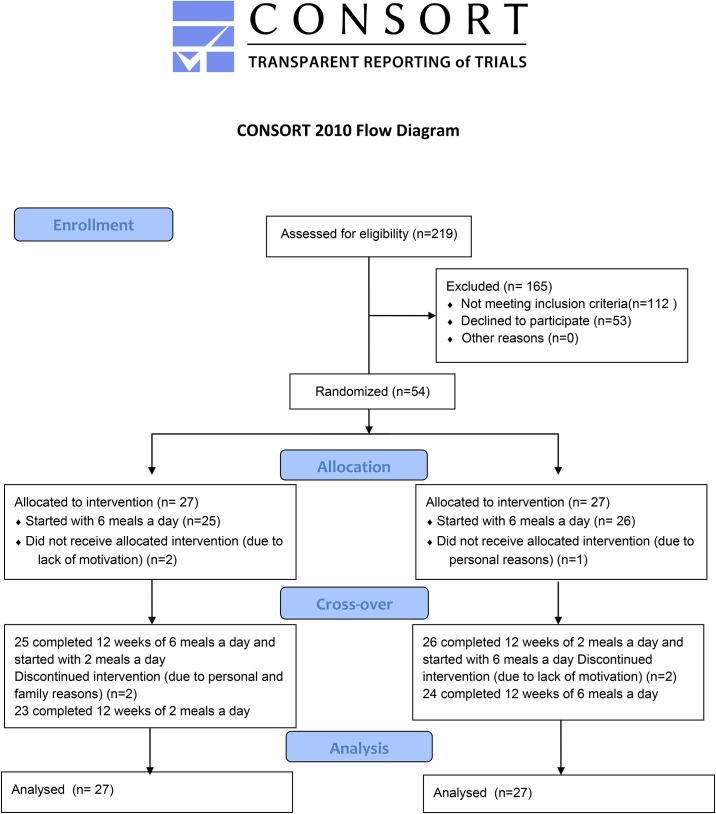
CONSORT flow diagram.

The protocol for this trial and supporting CONSORT checklist are available as supporting information; see S1–S3 Files. We included a group of 54 patients suffering from T2D (with disease duration of more than 1 year) treated by oral hypoglycaemic agents (both men and women), age 30–70 years, BMI 27–50 kg/m2 and HbA1c 6–11.8% (42–105 mmol/mol). Exclusion criteria comprised alcohol or drug abuse, pregnancy or lactation, unstable medication or weight in the last 3 months, a diagnosis of type 1 diabetes and the presence of a cardiostimulant. Written informed consent was obtained from all participants prior to enrollment in the study; the study protocol (Protocol S3), informed consent, and patient information were reviewed and approved by the Ethics Committee of the Thomayer Hospital and Institute for Clinical and Experimental Medicine in Prague, Czech Republic on December 18, 2009. On December 1, 2010 we started the telephone screening of potential participants. After approval of financial support, subjects were screened in person and enrolled into the study on January 10, and February 28, 2011. The first patient entered the intervention phase on March 14, 2011 and the last completed on October 20, 2011. In this single-center study the samples were collected in the Laboratory of Clinical Pathophysiology in Institute for Clinical and Experimental Medicine in Prague. There was no follow-up phase. The study flowchart is presented in Fig 1.

Of the 219 patients, who were screened, 54 participants were included. Randomisation and allocation to trial groups (n = 27 and n = 27) were carried out by a central computer system. Registration on ClinicalTrials.gov was initiated on January 6, 2011, after the telephone enrollment of participants started (Identifier: NCT01277471). The authors confirm that all ongoing and related trials for this intervention are registered. Characteristics of included participants, who underwent randomisation within a 24-week-crossover study, are presented in Table 1. After a 1-month-run-in-period, the participants started with a regimen of either six (A6) or two (B2) meals a day that was followed for 12 weeks. The A6 regimen consisted of three main meals (breakfast, lunch and dinner), and 3 smaller snacks in between. The B2 regimen consisted of breakfast (eaten between 6–10 a.m.) and lunch (eaten between noon and 4 p.m.). Then the participants switched regimens for a subsequent 12 weeks. All measurements were performed at weeks 0 (baseline), 12 and 24. The study protocol was approved by the Institutional Ethics Committee. After a 1-month-run-in-periodthe participants began a 12-week regimen of either six (A6) or two (B2) meals a day. The A6 regimen consisted of three main meals (breakfast, lunch and dinner), and three smaller snacks in between. The B2 regimen consisted of breakfast (eaten between 6–10 a.m.) and lunch (eaten between noon and 4 p.m.).

**Table 1:** Baseline characteristics of the study population.

Characteristic	Study group (n = 54)
Age—years	59.4±7.0
Sex—no. (%)	
Male	29 (54)
Female	25 (46)
Duration of diabetes—years	8.1±5.8
Smokers—no. (%)	10 (19)
Weight—kg	94.1±15.5
BMI—kg.m^-2^	32.6±4.9
HbA1c (DCCT)—%	7.2±3.3
HbA1c (IFCC)—mmol/mol	54.9±13.0
Systolic blood pressure—mm Hg	140±14
Diastolic blood pressure—mm Hg	85±8
Resting heart rate—beats.min^-1^	71±9
Oral hypoglycemic agents—no. (%)	
Metformin	41(76)
Sulfonylurea	16 (30)
Thiazolidinedione	3 (6)
Glinides	2 (4)
Acarbose	1 (2)
DPP-4 inhibitors	19 (35)
Lipid-lowering therapy—no. (%)	31 (57)
Antihypertensive therapy—no. (%)	33 (61)

Data are means ± SD.

### Diet

The diet composition in both regimens followed the DNSG (Study Group on Diabetes and Nutrition of the European Association for the Study of Diabetes) guidelines [30], and had the same caloric restriction of -500 kcal/day based on measurements of the resting energy expenditure of each subject by indirect calorimetry (metabolic monitor VMAX; Sensor Medics, Anaheim, CA, USA) [31]. The diet contained 50–55% of total energy from carbohydrates, 20–25% protein, less than 30% fat (≤7% saturated fat, less than 200 mg/day of cholesterol/day) and 30–40 g/day of fibre.

### Study procedure

All measurements were performed on an outpatient basis after overnight fasting with only tap water allowed ad libitum. The patients did not take any of their diabetes medication the evening or the morning before the assessments. Plasma concentrations of glucose, immunoreactive insulin, C-peptide, appetite and gastrointestinal (GI) hormones were measured at 0, 30, 60, 120, and 180 min after standard breakfast (453 kcal, 45% carbohydrates, 17% proteins, 38% lipids).

Compliance with both regimens was maximized by an initial 4-day tutorial at the beginning of each regimen where the patients learned in detail how to compose and divide their diet, with follow-up weekly meetings with lectures, cooking classes and food diary consultation with a registered dietician. Registered dieticians analysed 3-day dietary records (2 weekdays and 1 weekend day) completed by each participant at weeks 0, 12, and 24. Participants were asked not to alter their exercise habits during the study. Physical activity was monitored with a pedometer (Omron HJ-720IT, Omron, Kyoto, Japan), using the average 1- month step count for evaluation. The International Physical Activity Questionnaire [32] and the Baecke Questionnaire [33] were completed by each participant at weeks 0, 12, and 24. As described in detail previously [19].

### Analytical methods

The concentrations of GLP-1, GIP, PP, PYY, amylin, leptin and ghrelin were determined via multiplex immunoanalyses based on xMAP technology using a MILLIPLEX MAP Human Gut Hormone Panel (Millipore, Billerica, MA, USA) and a Luminex 100 IS analyzer (Luminex Corporation, Austin, TX, USA)[34]. Protease and dipeptidyl peptidase-4 inhibitors were added to two samples at each time point. The assay sensitivities, expressed as the minimum detectable concentrations reported in the instructions for use by the manufacturer, are (in pg/mL): amylin (total) 3,2; ghrelin 1.8; leptin 157.2; GIP 0.2; GLP-1 5.2; PP 2.4; and PYY 8.4. For our measurements, the sensitivity was set as the value of the lowest standard concentration.

### Statistical analyses

The power analysis was completed to estimate the number of subjects for the experiment with assumed weight loss (as the most important variable) for treated and untreated subjects 3 kg and 2 kg, respectively (assumed estimated difference between weight losses was 1 kg). The square root of the within mean square error was sw = 1 kg and effect size 1kg. The required probability of the positive probability of a false positive null hypothesis was α = 0.05 and the required number of subjects under investigation was 30, 24, 22, and 18 for power 0.96, 0.91, 0.88, and 0.8, respectively. Based on these data we considered the total number of 45 subjects as sufficient for the experiment (statistic software PASS 2005; Number Cruncher Statistical Systems, Kaysville, UT, USA). The intention-to-treat analysis included all participants. We tested the distributions of the data. If the distribution was skewed, we used the Box-Cox transformation to attain data symmetry and homoscedasticity [35]. 2x2 cross-over ANOVA (Statistical software Statgraphics Centurion XV version 15.2.06 from Statpoint Technologies, Inc., Herndon, VA, USA) was used for data evaluation. The model consisted of between-subject factors “sequence” and “gender”, the factor “subject”, and within-subject factors of “period and treatment”. We have checked the carry-over effect using the model including the factors “period” (order of diet regimens) and “sequence” and we have not found any significance of these factors for any dependent variables.

In a subsequent subanalysis, to estimate the postprandial changes, repeated-measures ANOVA was performed. The factors of “subject”, “sequence”, “diet” and “time” were included in the analyses. In each “sequence” the interactions between “diet” and “time” (“diet” x “time”) were calculated for each variable. The data are presented as the means with 95% confidence intervals. We have checked the carry-over effect using the model including the factor “sequence” and we have not found any significance of this factor for any dependent variables.

Spearman's correlations were calculated for the relationship between changes in concentrations of investigated parameters. They were compared in 4 periods (0–30, 30–60, 60–120, 120–180 minutes after ingestion of standard breakfast).

## Results

**Fig 2:**
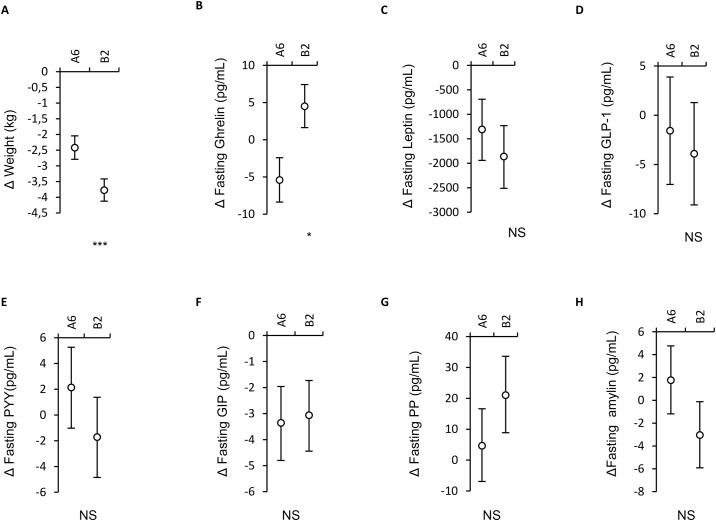
Changes in anthropometric and laboratory parameters. Data are shown as changes from baseline in response to the regimen of six (A6) and two meals a day (B2). Data are means with 95% CI. Significance of the factor treatment (assessed by 2x2 crossover ANOVA) is indicated by * for p<0.05, ** for p<0.01, *** for p<0.001 and NS for non-significant. a: Δ Weight, n = 54, b: Δ Fasting ghrelin, n = 54, c: Δ Fasting leptin, n = 54, d: Δ Fasting GLP-1, n = 54, e: Δ Fasting PYY, n = 54, f: Δ Fasting GIP, n = 54, g: Δ Fasting PP, n = 54, h: Δ Fasting amylin, n = 54.

The results are expressed as the changes in response to the A6 and B2 diet regimens. They are presented as means with 95% confidence intervals in Fig 2.

**Fig 3:**
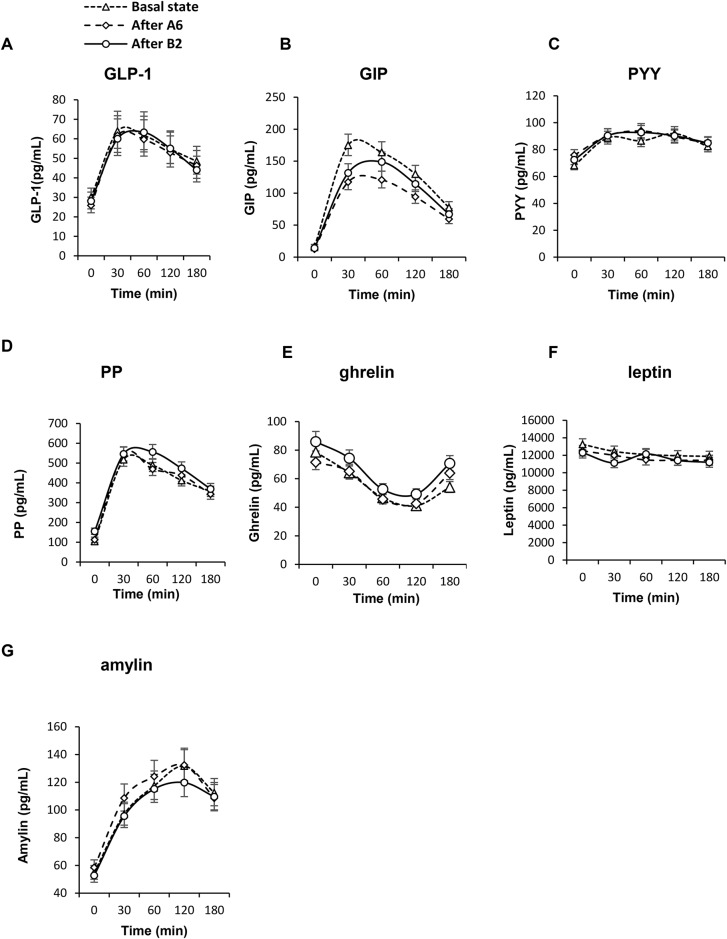
Postprandial changes in plasma concentrations of gastrointestinal and appetite hormones after standard meal ingestion. At basal state (triangle, dotted line), after the diet regimen with six meals a day, A6 (rhombus, dashed line) and after the diet regimen with 2 meals a day, B2 (circle, dashed line). Data are expressed as means with 95%CI. **A: GLP-1**: Factors diet: F = 0.4, p = 0.6828; time: F = 28.5, p<0.0001; subject: F = 37.5, p<0.0001; interaction diet × time: F = 0.1, p = 0.9984. **B: GIP**: diet: F = 17.3, p<0.0001; time: F = 291.7, p<0.0001; subject: F = 17, p<0.0001; interaction diet × time: F = 0.7, p = 0.7093.**C: PYY**: diet: F = 1.7, p = 0.1897; time: F = 20.7, p<0.0001; subject: F = 29.1, p<0.0001; diet × time: F = 0.6, p = 0.8173. **D:PP**: diet: F = 8.7, p = 0.0002; time: F = 298, p<0.0001; subject: F = 101.4, p<0.0001; interaction diet × time: F = 1.1, p = 0.3409. **E: ghrelin:** diet: F = 13.6, p<0.0001; time: F = 61.6, p<0.0001; subject: F = 47.5, p<0.0001; interaction diet × time: F = 0.9, p = 0.503. **F:leptin:** diet: F = 3.7, p = 0.0263; time: F = 3.6, p = 0.0074; subject: F = 274, p<0.0001; interactiondiet × time: F = 0.5, p = 0.8241. **G: amylin**: diet: F = 1.9, p = 0.1551; time: F = 83, p<0.0001; subject: F = 27, p<0.0001; interactiondiet × time: F = 0.3, p = 0.9669.

The postprandial profiles of appetite and gastrointestinal hormones measured at 0, 30, 60, 120, and 180 min after the meal at basal state and after the A6 and B2 diet regimens are illustrated in Fig 3.

### Body weight

Body weight decreased under both regimens more with B2 (*p* = 0.0005; −2.4 kg; 95% CI −2.8, −2.0 kg with A6 vs. −3.8 kg; 95% CI −4.1, −3.4 kg with B2) as already described previously [19]. As far as gender differences are concerned, the body weight at baseline in men vs. women (*p* = 0.99) and also the body weight reduction after the diet regimens (*p* = 0.87; -3.0 kg;95% CI -3.9, -2.2 kg in women vs. -3.2 kg; 95% CI -3.9, -2.4 kg in men) were similar.

### Appetite hormones

Fasting leptin decreased comparably in both regimens (*p* = 0.37; -1308pg/ml; 95% CI: - 1941, -693pg/ml with A6 vs. -1862; 95% CI: -2513, -1231pg/ml with B2). In women, the fasting concentrations of leptin were significantly higher than in men (*p* = 0.000003). If we consider the factor “gender” in the statistical model the hypocaloric diet reduced significantly leptin but only in females. In males the change in fasting leptin was not significant (*p* = 0.02; -3000 pg/ml; 95% CI: -4423, -1672pg/ml in women vs. -255 pg/ml; 95% CI: -1174, 626pg/ml in men). Fasting plasma levels of ghrelin decreased in A6 and increased in B2 with significant difference between both diet regimens (*p* = 0.023;-5.42 pg/ml; 95% CI:-10.1, -0.1pg/ml with A6 vs. 4.5pg/ml; 95% CI: 0.37, 8.5 pg/ml with B2). In a subsequent subanalysis, the decrease in body weight correlated negatively with changes in fasting ghrelin (r = -0.4, *p*<0.043). The postprandial drop in ghrelin levels correlated positively with its fasting level (*r* = 0.9, *p*<0.001). Reduced feelings of hunger after the hypocaloric diet regimen with B2 were previously described in detail elsewhere. In the added subanalysis, fasting plasma ghrelin levels did not correlate with changes in hunger (p = 0.7). The changes in fasting concentrations of ghrelin were similar in men vs. women (*p* = 0.85; -1.36pg/ml; 95% CI:-13.29, 9.8pg/ml in women vs. 0.59 pg/ml; 95% CI:-8.5, 9.2pg/ml in men). The two different diet regimens induced relatively similar postprandial responses of the both appetite hormones, leptin and ghrelin, when the time-course (interaction diet x time) is considered (*p* = 0.8241and *p* = 0.503, respectively) (Fig 3E and 3F). The plasma concentrations of ghrelin were significantly higher at fasting state and after 180 minutes after meal ingestion after the diet regimen B2.

### Gastrointestinal hormones

Fasting GIP decreased comparably in both regimens (*p* = 0.83; -3.36; 95% CI: -4.8, -1.96pg/ml with A6 vs.-3.1; 95% CI: -4.44, -1.73 pg/ml with B2;) and also comparably in men and women (*p* = 0.39). During the postprandial phase, there was a considerable difference in the GIP response at basal state and after both diet regimens (*p*<0.0001) (Fig 3B). There was a significantly higher postprandial GIP response at baseline in first 30 minutes after the meal ingestion than after both hypocaloric diet regimens, where the postprandial responses were similar. The postprandial curves of GIP have the same shapes in time in all three measurements (interaction diet × time: *p* = 0.7093). Fasting PP increased in B2 and did not significantly changed in A6 with no significant difference between both regimens (*p* = 0.17; 4.64 pg/ml; 95% CI: -6.9, 16.6 pg/ml with A6 vs. 21.0 pg/ml; 95% CI: 8.87, 33.6pg/ml with B2). Gender differences in changes of fasting levels of plasma PP concentrations were also not significant (*p* = 0.76). When the time-course is considered, the postprandial responses of PP were similar with rapid increase in the first 30 minutes and then slower fall (interaction diet x time: *p* = 0.34) (Fig 3D). GLP-1 did not change after any regimen (*p* = 0.65; -1.571 pg/ml; 95% CI: -7.02, 3.88 pg/ml with A6 vs. -3.91 pg/ml; 95% CI: -9.1, 1.28 pg/ml with B2), with no difference in gender (*p* = 0.39). The postprandial responses of GLP-1 were similar in all three meal tests (interaction diet x time: *p* = 0.998) (Fig 3A).

The fasting plasma levels of PYY did not change (*p* = 0.21; 2.14; 95% CI: -1.03, 5.26pg/ml with A6 vs. -1.71; 95% CI: -4.84, 1.37pg/ml with B2) with no difference in men vs. women (*p* = 0.79). The postprandial responses of PYY were also similar as illustrated in Fig 3C.

Fasting amylin decreased after B2 and did not significantly change after A6 with no significant difference between both diet regimens (*p* = 0.1; 1.77pg/ml; 95% CI:-1.18, 4.76pg/ml in A6 vs. -3.04; 95% CI: -5.91, -0.12pg/ml in B2). Gender differences in changes of fasting levels of amylin plasma concentrations were also not significant (*p* = 0.9). The postprandial responses of amylin were similar in all three meal tests (interaction diet x time: *p* = 0.97) (Fig 3G).

### Correlations of postprandial concentrations of Δ glucose, Δ IRI and Δ c-peptide, Δ GIHs and Δ appetite hormones

Postprandial secretion of measured gastrointestinal hormones was increased in parallel with glucose and insulin concentrations as shown in Table 2. In the first 30 minutes after meal ingestion (0–30´), a positive relationship was found between Δ PYY and Δ glucose, Δ Immunoreactive insulin (IRI) and Δ c-peptide. Changes in PYY concentrations were also correlated with changes in leptin, GIP (*p* = 0.035, *p* = 0.016, respectively) and strongly correlated with changes in GLP-1, amylin and PP (*p*<0.001 all). Δ GLP-1 correlated also with ΔIRI (*p* = 0.019). Changes in amylin concentrations correlated with changes in glucose (*p* = 0.003), leptin (*p* = 0.002) and all measured GIH´s. Between 30–60 minutes after meal ingestion a positive relationship was found between Δ GLP-1 and Δ glucose (*p* = 0.004) and Δ amylin and Δ glucose (*p* = 0.044). Δ leptin correlated with ΔGIP (*p* = 0.029). Changes in GLP-1 concentrations correlated with changes in GIP (*p* = 0.015), amylin, PP (*p* = 0.007, *p* = 0.001, respectively) and PYY (p<0.001) concentrations. Between 60–120 minutes after meal ingestion changes in amylin correlated positively with ΔPYY and GLP-1 (*p* = 0.001, *p*<0.001, respectively). Changes in amylin concentrations correlated strongly with changes in glucose, IRI and c-peptide (*p*<0.001 all). The correlations between Δ GLP-1, Δ GIP, Δ PYY and Δ PP remained significant. Between 120–180 minutes after meal ingestion there was an inverse correlation between Δ ghrelin and Δ glucose (*p* = 0.026). A positive relationship was found between Δ IRI, Δ GLP-1 and ΔGIP (*p* = 0.008, *p* = 0.005, respectively). Changes in GIP concentrations strongly correlated with changes in Δ GLP-1, amylin, PP and PYY (p<0.001 all).

**Table 2:** Correlations of postprandial concentrations of Δ glucose, Δ IRI and Δ c-peptide, Δ GIH´s and Δ appetite hormones, n = 54.

**Δ (0–30)´**	**Δ glucose**	**p**	**Δ IRI**	**p**	**Δ C-peptide**	**p**	**Δ GIP**	**p**	**Δ GLP-1**	**p**	**Δ amylin**	**p**	**Δ PP**	**p**	**Δ PYY**	**p**
**Δ ghrelin**	-0.209		-0.104		-0.080		0.079		0.082		0.020		0.082		0.183	
**Δ leptin**	-0.135		-0.009		0.030		0.041		0.127		**0.339**	**	0.130		**0.236**	*
**Δ GIP**	0.138		0.104		0.190				**0.331**	**	**0.340**	**	0.214		**0.269**	*
**Δ GLP-1**	0.104		**0.261**	*	0.160		**0.331**	**			**0.381**	***	**0.415**	***	**0.579**	***
**Δ amylin**	**0.326**	**	0.209		0.176		**0.340**	**	**0.381**	***			**0.307**	**	**0,440**	***
**Δ PP**	0.089		0.138		0.076		0.214		**0.415**	***	**0.307**	*			**0.420**	***
**Δ PYY**	**0.237**	*	**0.236**	*	**0.281**	*	**0.269**	*	**0.579**	***	**0.440**	***	**0.420**	***		
**Δ (30–60)´**	**Δ glucose**	**p**	**Δ IRI**	**p**	**Δ C-peptide**	**p**	**Δ GIP**	**p**	**Δ GLP-1**	**p**	**Δ amylin**	**p**	**Δ PP**	**p**	**Δ PYY**	**p**
**Δ ghrelin**	-0.213		-0.105		-0.151		-0.055		-0.048		-0.154		0.106		-0.027	
**Δ leptin**	0.026		-0.192		-0.082		**0.250**	*	0.065		0.072		0.144		0.213	
**Δ GIP**	0.084		0.048		0.146				**0.278**	*	0.181		**0.274**	*	**0.334**	**
**Δ GLP-1**	**0.330**	**	0.165		0.175		**0.278**	*			**0.308**	**	**0.364**	***	**0.423**	***
**Δ amylin**	**0.232**	*	0.103		0.159		0.181		**0.308**	**			0.050		**0.580**	***
**Δ PP**	0.120		0.050		0.010		**0.274**	*	**0.364**	***	0.050				0.159	
**Δ PYY**	0.094		0.075		0.169		**0.334**	**	**0.423**	***	**0.580**	***	0.159			
**Δ (60–120)´**	**Δ glucose**	**p**	**Δ IRI**	**p**	**Δ C-peptide**	**p**	**Δ GIP**	**p**	**Δ GLP-1**	**p**	**Δ amylin**	**p**	**Δ PP**	**p**	**Δ PYY**	**p**
**Δ ghrelin**	-0.030		-0.086		0.033		0.036		-0.027		0.009		0.112		0.051	
**Δ leptin**	0.142		-0.202		**-0.234**	*	**0.320**	**	0.007		-0.103		**0.268**	*	0.095	
**Δ GIP**	**0.262**	*	0.094		-0.026				**0.378**	***	**0.389**	***	**0.357**	***	**0.487**	***
**Δ GLP-1**	0.134		0.117		0.033		0.378	***			**0.455**	***	**0.296**	**	**0.735**	***
**Δ amylin**	**0.437**	***	**0.448**	***	**0.443**	***	0.389	***	0.455	***			0.118		**0.382**	***
**Δ PP**	0.077		-0.088		-0.072		0.357	***	0.296	**	0.118				**0.241**	*
**Δ PYY**	0.155		0.085		0.051		0.487	***	0.735	***	0.382	***	0.241	*		
**Δ (120–180)´**	**Δ glucose**	**p**	**Δ IRI**	**p**	**Δ C-peptide**	**p**	**Δ GIP**	**p**	**Δ GLP-1**	**p**	**Δ amylin**	**p**	**Δ PP**	**p**	**Δ PYY**	**p**
**Δ ghrelin**	**-0.240**	*	-0.103		-0.176		-0.091		-0.097		-0.040		-0.002		0.098	
**Δ leptin**	-0.092		0.026		0.079		-0.029		0.001		-0.008		-0.039		-0.043	
**Δ GIP**	**0.267**	*	**0.297**	**	0.178				**0.424**	***	**0.338**	***	**0.467**	***	**0.445**	***
**Δ GLP-1**	0.160		**0.284**	**	0.165		**0.424**	***			-0.070		0.195		**0.581**	***
**Δ amylin**	0.065		0.033		**0.269**	*	**0.338**	***	-0.070				0.135		0.159	
**Δ PP**	0.159		0.119		0.116		**0.467**	***	0.195		0.135				**0.294**	**
**Δ PYY**	-0.021		0.156		0.066		**0.445**	***	**0.581**	***	0.159		**0.294**	**		

* denote p < 0.05,

** denote p < 0.01,

*** denote p < 0.001.

## Discussion

### Appetite hormones

In this secondary analysis, we tested the effect of two hypocaloric diet regimens with different meal frequencyon fasting and postprandial levels of appetite and GI hormones. We have shown previously that in patients with type 2 diabetes breakfast and lunch consumption reduced feelings of hunger [20] more than a diet with the same caloric restriction divided into six more frequent meals. Our results indicate that lower meal frequency in a reduced-energy regimen, leading to greater reduction in body weight, is also associated with an increase in fasting plasma levels of ghrelin, that negatively correlate with changes in body mass index. This is a very good outcome given the fact that it may increase hunger after waking up, encouraging the patients to eat more calories for breakfast. Obese and T2D individuals usually have low hunger sensation at wake up, making difficult to eat breakfast. Moreover, the breakfast omission is associated with increased risk of poor glycaemic control and visceral adiposity despite the same daily energy intake [36–38]. The rise in ghrelin plasma levels in B2 group is also likely the result of longer fasting period between both meals, that will be in line with the results of a study suggesting that the preprandial increase in ghrelin is associated with inter-meal interval [39]. The variations of body weight lead to compensatory responses of fasting ghrelin levels. Ghrelin is known to be reduced in obese subjects and increased after diet-induced weight loss [25]. It is supposed to stimulate appetite and food intake, enhancing fat mass deposition and weight regain. However, the physiological importance of ghrelin as a regulator of energy homeostasis is still unclear, because there are also studies, which do not confirm the association between weight loss following the hypocaloric diet and the increase in fasting ghrelin [40]. There is some evidence that the increase of fasting plasma concentration of ghrelin appear to be correlated with the increase [41, 42], but also the decrease in hunger [43]. In our study the changes in fasting plasma ghrelin levels did not correlate with changes in hunger. In one randomized controlled study, less frequent eating was related to increased satiety and appetite control during the day [12]. Decreased hunger and increased satiety in response to lower meal frequency has already been demonstrated earlier [44]. One probable explanation of this finding is that larger meals impart the sense of fullness and satiety and decreased postprandial ghrelin levels [12], making periods of fasting more bearable than being hungry all day, from eating more smaller meals without getting full and being in the postprandial phase for the majority of the day. In lean healthy individuals, the ghrelin plasma concentrations increase during fasting and are suppressed by meal intake [45]. In obese subjects and patients with T2D, ghrelin secretion is down-regulated and the decline in plasma ghrelin after a meal is diminished [22, 23]. Weight loss induced by food restriction increases ghrelin plasma levels [24, 25] and improves the postprandial ghrelin response independently of diet composition [26]. Postprandial decreases in ghrelin were also positively correlated with inter-meal interval in healthy normal weight subjects. It was shown in one study that meal request after energy restriction was preceded by an increase in ghrelin and was positively correlated with inter-meal interval most profound after energy restriction, not during energy balance diet [39]. Periods of fasting between meals may be even more important than the composition of the diet. As far as the postprandial state is concerned, the plasma concentrations of ghrelin were significantly higher at fasting state and after 180 minutes after meal ingestion after the diet regimen B2. We described the positive association between the baseline level of ghrelin and its decrease after meal ingestion. It means that the higher the baseline level of ghrelin, the larger the postprandial drop. Furthermore, between 120–180 minutes postprandially, there was an inverse correlation observed between changes in plasma ghrelin levels and changes in plasma glucose levels. The increase of ghrelin preprandially might be dependent on the return of plasma glucose to fasting concentrations. The failure of returning postprandial glucose plasma levels to normal levels causes a lack of a preprandial increase in ghrelin concentration. That is in accordance with previous findings [39].

In our study, fasting leptin decreased in response to both regimens with no difference between the treatments. Leptin, an adipokine secreted from white adipose tissue, is known for its effects on decreasing appetite and food intake. Obesity and T2D are associated with increased leptin levels and resistance to leptin action [46]. Increased leptin levels, probably reflecting leptin resistance, were strongly related to insulin resistance [47]. The two different diet regimens induced expected similar postprandial responses of leptin, which acts rather as a long-term adiposity signal than a gastrointestinal signal that regulates postprandial state [1].

The fasting concentrations of leptin at baseline were significantly higher in women than in men. According to previous findings, plasma leptin concentration is directly related to the degree of obesity and is higher in women than in men of the same body mass index [48, 49]. Furthermore, according to our results, the hypocaloric diet reduced significantly leptin but only in females. In males the leptin concentrations remained unchanged. The weight at baseline in men vs. women and also the body weight reduction after the diet regimens were similar. Also in a study with 6-month weight loss intervention, relative decline in circulating leptin was greater in women than in men, although the percentage of fat mass loss was similar [50]. Whereas that leptin is believed to increase energy expenditure [51], this sex asymmetry is supposed to represent an evolutionary paradigm for females to resist the loss of energy stores [52].

### Amylin

Fasting amylin decreased in the hypocaloric diet regimen with lower frequency of meals. It is known to regulate both glucose and energy homeostasis [53]. Proportional changes in amylin and insulin concentrations were highly correlated, because amylin is a peptide co-secreted with insulin upon glucose stimulation [54]. It also suppresses gastrointestinal motility and food intake [55]. In obese humans, the amylin analog pramlintide elicited sustained reductions in food intake and body weight [56]. Amylin seems to be particularly effective when combined with other hormones such as leptin. According to our study, the postprandial responses of amylin were similar in all three meal tests. Postprandial changes in amylin concentrations correlated with changes in concentrations of all measured GIHs, and there was a strong correlation with postprandial changes of leptin. Leptin is known as a long-term adiposity signal, whereas gastrointestinal hormones, such as amylin are regarded as short-term satiety signals. There might be a potential interaction between them and according to some evidence amylin agonism could even restore leptin responsiveness in diet-induced obesity [57]. It is worth noting that amylin interacts with numerous gastrointestinal hormones to control eating and mediate the eating inhibitory effect of some of these hormones, most prominently peptide YY and GLP-1 [58]. These combinations lead to a stronger reduction of eating control than single hormones alone.

### Glucagon- like peptide-1, peptide YY and pancreatic polypeptide

In the present study, fasting plasma levels of neither GLP-1 nor PYY and also their postprandial responses did change after neither hypocaloric diet regimens. Despite we have expected lower fasting plasma levels and higher postprandial responses after both diet regimens more pronounced with B2, because of bigger weight loss. It was demonstrated that both peptides exert anorexigenic effects and that their satiating effects should be even additive [28]. Caloric restriction and body weight reduction are supposed to decrease fasting plasma levels [7, 8, 10] and increase meal responses of PYY and GLP-1 [9]. Studies with hypocaloric diet regimens indicate that lower meal frequency should also have positive effect on GLP-1s postprandial response [12]. From some studies in obese people after gastric bypass surgery is evident, that postprandial response of both peptides is significantly higher with larger weight loss [59]. On the contrary, another study suggests that it is not the weight loss, but rather the surgical procedure, which increases GLP-1 levels [60]. In humans, intravenous administration of GLP-1 increases satiety, inhibit gastric emptying and decreases weight [61]. Obese individuals and patients with T2D have been reported to have delayed postprandial release of GLP-1 with improvement after weight loss [62] and that successful weight loss maintenance includes long-term increase meal responses of GLP-1 and PYY [9]. Correlation of changes in GLP-1 and insulin during the whole postprandial phase observed by the authors is not surprising due to its well-documented incretin effect.

PYY plays an important regulatory role in GIT function [63]. It is produced in all segments of the intestine and co-secreted predominantly from the endocrine L cells in the ileum together with GLP-1. The role of PYY in feeding regulation has attracted considerable attention. In humans it has been reported, that it is an anorectic hormone, because itdecreased food intake and appetite when injected peripherally [3]. The level of circulating PYY may also be increased by exercise [64]. It was demonstrated that PYY levels are decreased by complete fasting for more than two days in lean subjects and it was not regulated by leptin [10]. In obese state, the fasting and postprandial PYY levels are often lower [65], but not all studies have confirmed this to be true [66]. In contrast to reduced sensitivity to leptin among obese and diabetic patients, the sensitivity to the anorectic effects of PYY remained significant [67]. Postprandially, we have not found any effects of both hypocaloric diet regimens on the response after the standard meal. According to previous studies, the initial postprandial response of PYY is supposed to be attenuated in patients with T2D [6], especially after a fatty meal [68]. The increased PYY3–36 response represents an improved capacity to regulate satiety and potentially body weight in insulin-resistant patients [11]. We were expecting an increase in meal-stimulated PYY response after B2.

We have found a positive relationship between postprandial changes in PYY and insulin. To the best knowledge of the authors, the association between PYY and postprandial insulin levels have not been published yet.

As far as PP is concerned, we have detected the increase in fasting PP after B2with no significant difference between both diet regimens. Its role in patients with T2D is uncertain. Some studies have shown higher fasting levels with blunted postprandial response, similar to PYY [68]. According to previous studies, peripheral administration of PP reduced food intake in lean healthy individuals [27]. We have not found any effects of both hypocaloric diet regimens on the postprandial response of PP. According to a recent overview, the effects of a hypocaloric regimen or changes in body weight on fasting or postprandial levels of PP were not proven [29].

### GIP

We detected that fasting plasma levels of GIP decreased comparably after both hypocaloric diet regimens. The reduction in fasting plasma concentration following a hypocaloric diet is a positive finding, because increased GIP levels directly promote fat storage and energy deposition [69]. It is released postprandially in response to feeding, especially with high fat diet. It produces a glucose-dependent stimulation of insulin secretion consistent with its role as an incretin hormone [70]. We detected the positive correlation of changes in GIP and insulin between 120–180 minutes after meal ingestion. It may be interpreted that beta-cells in T2D are insensitive to meal and stimulation by incretin hormone GIP in the early postprandial period. This is in accordance with previous findings [71].

Postprandially, there was a significantly higher GIP response at baseline in first 30 minutes after the meal ingestion than after both hypocaloric diet regimens, where the postprandial responses were similar. We have expected lower postprandial response in the regimen B2 due to a bigger weight loss, which is supposed to mediate this effect [72]. In one intervention study hypocaloric diet following a weight loss was associated with reduction in GIP response to ingested glucose [11]. Diet regimens with calorie reduction that lower the postprandial response of the anabolic hormone GIP could be beneficial and effective for weight management [73]. It has been suggested that changes in insulin secretion following a lifestyle intervention might be mediated via alterations in GIP secretion [74].

Several studies have shown that an increase in meal size increases diet-induced thermogenesis (DIT). It has been demonstrated that a large isocaloric mixed meal causes a greater postprandial thermogenic response than the same food consumed in six smaller portions [75]. It was also demonstrated, that lower postprandial response of GIP leads to greater postprandial thermogenesis and that GIP response could be a negative regulator of postprandial thermogenic efficiency [73]. In current study we have not investigated postprandial thermogenesis and possible correlations with gastrointestinal hormone release or the effect of caloric restriction or meal frequency on DIT. This mechanism could explain the greater weight loss with the B2 regimen.

The study has a number of strengths. First of all, the cross-over design increased our power to detect differences in response to both regimens. Second, we used a physiological approach to measure the concentrations of GIHs in both fasting and postprandial state after the ingestion of a standardized meal.

The main limitation of our study is the relatively short duration, which precludes a generalisation of our findings. Also, the duration of diabetes in our patients was quite short. All patients were treated by oral hypoglycemic agents, so the sample of diabetic patients in our study was not representative of the whole T2D population. Lower caloric intake leading to bigger weight loss in B2 could be a confounding factor of the results. We have to admit the possibility of a reduced energy intake with the B2 regimen, even though the energy intake reported by our participants was similar in both regimens. Reported dietary intake and also physical activity were comparable in both regimens. Another limitation is the number of statistical tests and factors in the statistical model, which may cause inaccurate findings. A further limitation is that the present study revealed interrelation ships, not causal relationships and we cannot therefore address the mechanisms underlying the correlations.

## Conclusions

Both hypocaloric diet regimens decreased fasting leptin and GIP and reduced the postprandial response of GIP similarly. Eating only breakfast and lunch increased fasting plasma ghrelin more than the same caloric restriction split into six meals. The changes in fasting ghrelin correlated negatively with the decrease in body weight. These results suggest that for type 2 diabetic patients on a hypocaloric diet, eating larger breakfast and lunch may be more efficient and therefore beneficial than six smaller meals during the day.

## Supporting information

S1 File Checklist. (DOC)

S2 File Study protocol in English. (DOC)

S3 File Study protocol in Czech. (DOC)
