# Supplementary material for: The effect of meal frequency in a reduced-energy regimen on the gastrointestinal and appetite hormones in patients with type 2 diabetes: A randomised crossover study
Source: PLoS One. 2017 Apr 3;12(4):e0174820. doi: 10.1371/journal.pone.0174820 (PMC5378398; doi:10.1371/journal.pone.0174820)
Supplement: S2 File — (DOC) [file pone.0174820.s002.doc]

**The Effect of Meal Frequency in a Reduced-energy Regimen on Insulin Resistance, Insulin Secretion, Hepatic Fat Content, Gastrointestinal and Appetite Hormones in Subjects with Type 2 Diabetes**

Introduction

Insulin resistance is very likely to play role in developing type 2 diabetes and wide range of clinical, biochemical and humoral abnormalities, which we call metabolic syndrome. Efforts to reduce insulin resistance are a treatment pillar not only of type 2 diabetes, but of the whole syndrome. Regimen changes i.e. lowering diet and increased physical activity are surely basic treatment methods. In diet besides caloric restriction also frequency of meals may be important. At present the issue of whether it is healthier to eat small frequent meals or less-frequent larger meals is currently being discussed.

The size and frequency of meals are fundamental aspects of nutrition that can have profound effects on the health and longevity of laboratory animals. In humans, excessive energy intake is associated with increased incidence of cardiovascular disease, diabetes, and certain cancers and is a major cause of disability and death in industrialized countries. On the other hand, the influence of meal frequency on human health and longevity is unclear. Both caloric restriction and reduced meal frequency/intermittent fasting can suppress the development of various diseases and can increase life span in rodents by mechanisms involving reduced oxidative damage and increased stress resistance.

Many of the beneficial effects of caloric restriction (CR) and fasting appear to be mediated by the nervous system. For example, intermittent fasting results in increased production of brain-derived neurotrophic factor (BDNF), which increases the resistance of neurons in the brain to dysfunction and degeneration in animal models of neurodegenerative disorders; BDNF signaling may also mediate beneficial effects of intermittent fasting on glucose regulation and cardiovascular function (Mattson 2005b).

Animal studies strongly support antidiabetic effects of CR and intermittent fasting diets. Intermittent fasting regimens, such as every-other-day fasting (EODF, the most commonly employed protocol in animal studies), can increase the life span of rats and mice (Carlson and Hoelzel 1946, Goodrick et al. 1982). Both CR and EODF reduce blood glucose and insulin concentrations and improve glucose tolerance in rodents (Anson et al. 2003, Wang et al. 1997). The issue of whether it is healthier to eat small frequent meals or less-frequent larger meals is currently the subject of considerable debate (Mattson 2005a). Although it has been reported that patients with diabetes are better able to “control” their blood glucose concentrations when they eat regular small meals, an intermittent very-low-calorie diet improved weight loss and glycemic control more effectively than did moderate CR in subjects with type 2 diabetes (Williams et al. 1998). Moreover, two different fasting regimens (EODF and fasting two days per week) reduced diabetes incidence in Brattleboro rats (Pedersen et al. 1999). Increased insulin sensitivity appears to be the major mechanism responsible for the antidiabetic effects of energy restriction and intermittent fasting (Granberry and Fonseca 1999, Anson, Jones, and de Cabod 2005).

Epidemiological data suggesting benefits of increased meal frequency on CHD risk and lipid and lipoprotein levels in healthy subjects may be flawed because of biases associated with the method of data collection (Mann 1997). Studies with subjects with type 2 diabetes are very limited both in length and in number of subjects. A longer-term study, comparing the effect of three and nine meals daily (each period lasted four weeks), in 13 subjects with type 2 diabetes, did not confirm the potential benefits of increased meal frequency in type 2 diabetes (Arnold, Mann, and Ball 1997). Further larger-scale, long-term studies are essential before offering recommendations in terms of meal frequency.

Aims and priorities of the project

The purpose of this study is to

1) test the effect of frequency of meals (six vs. two meals daily with the same daily caloric restriction of -500 kcal/day) on insulin sensitivity, insulin secretion, and hepatic fat content.

2) characterize some of the mechanisms of action of different frequencies of meals (amount of visceral fat, hepatic fat content, serum concentrations of adipokines, gastrointestinal and appetite hormones, oxidative stress markers).

3) test the ability of the participants to maintain hypocaloric diet on both regimens when educated and left to prepare their meals alone in comparison with those for whom all meals during the study will be provided.

It will be a randomized, crossover study, where 50 individuals with type 2 diabetes will change in a random order two regimens: six, and two meals a day. Each testing period will take three months.

Glucose and lipid metabolism and its regulation will be thoroughly tested at start, and after each 3-months-period (meal test, hyperinsulinemic isoglycemic clamp, indirect calorimetry, MRI scan of the liver, DXA scan, serum concentration determination of selected adipokines, gut hormones, and oxidation stress markers).

Hypothesis

We hypothesize that low plasma insulin levels (as achieved by periods of fasting) will reduce insulin resistance and hepatic lipid content. In contrast, frequent meals (and consequent higher plasma levels of insulin) will predispose to non-alcoholic fatty liver disease and insulin resistance. Lower meal frequency will be associated with increased plasma levels of glucagon-like peptide -1 (GLP-1), peptide YY (PYY) and pancreatic polypeptide (PP) and higher postprandial reduction of ghrelin. Further lower meal frequency will be associated with lower oxidative stress markers.

We further hypothesize that the participants will increase their caloric intake with increased meal frequency (in spite of thorough education) when left to prepare their meals in comparison with those for whom all meals will be provided.

Key words

Insulin resistance, meal frequency, type 2 diabetes mellitus, non-alcoholic fatty liver disease, adipokines, gastrointestinal hormones, appetite hormones, oxidative stress

Methodology

Study design: we will use the design of a randomized, crossover study, where 50 individuals with type 2 diabetes will change in a random order the frequency of their meals: six, and two meals a day. Caloric restriction will be the same (-500 kcal/day). Each testing period will take three months. For one half of the participants all meals during the study will be provided. The other half will be thoroughly educated how to maintain their daily caloric intake during both regimens and they will prepare their meals alone.

Study group: 50 individuals with type 2 diabetes treated by diet only or oral hypogylycemic agents, diabetes duration at least 1 year, both men and women, age 30-65 years, BMI 27-50 kg/m². The subjects will be explained aims, methods and risks of the study and they will sign informed consent (Appendix 1).

Regimens: On the six-meals-per-day-regimen, participants will be asked to divide their total caloric intake into six meals and to eat every two or three hours. On the two-meals-per-day-regimen, they will divide their total caloric intake into two meals: the first meal will be eaten between 6 and 10 a.m., the second one between noon and 4 p.m.

Physical activity: Participants will be asked not to change their exercise habits during the study. Physical activity will be monitored using pedometers and standardized questionnaires: International Physical Activity Questionnaire (IPAQ), and Baecke questionnaire.

Procedures:

At the beginning (week 0), and at the end of every 3 months (week 12, and 24), the following procedures and measurements will be performed at each subject (three times in each subject):

1. Common anthropometric investigations (body weight, BMI, waist and hip circumference), blood samples will be taken for laboratory assessments (common laboratory tests, parameters of glucose and lipid metabolism, chosen adipokines, oxidative stress markers, gastrointestinal peptides, fatty acid composition in serum phospholipids etc.- see analytic methods).

2. Meal test for glucose tolerance assessment after standard breakfast /baguette Crocodille Cheese Gourmet - 180g, energy 452,8 Kcal/1895,7 kJ, composition: carbohydrates 49,2 g (44,55%), proteins 18,5 g (16,74%), lipids 18,8 g (38,7%), of which saturated 6,8 g, monounsaturated 6,0 g, polyunsaturated 5,0 g/. Blood samples for the assessment of glycemia, C- peptide, immunoreactive insulin (IRI) will be taken in 0, 30, 60, 120 and 180 minutes after breakfast.

3. Hyperinsulinemic (1 mU/kg/min) isoglycemic clamp (HIC) 3 hours long with indirect calorimetry. This method allows exact quantification of insulin resistance and energy substrates utilisation.

4. MRI (magnetic resonance imaging) scan of the liver to measure the hepatic fat content.

5. DXA (Dual energy X-ray absorptiometry) scan to measure total body composition and fat content.

Analytic methods:

Plasma concentrations of selected adipokines (resistin, adiponectin total, HMW- adiponectin, TNFalfa and leptin) and other cytokines and proteins (FABP) will be measured enzymatically using standard kits (ELISA, Linco, USA). Plasma levels of gut hormones will be measured enzymatically usig standard kits (Milliplex, Millipore, USA). Parameters of lipid peroxidation will be determined according to the levels of TBARS by the reaction with thiobarbituric acid and according to the levels of conjugated dienes.

The level of reduced and oxidised glutathione will be determinated using HPLC method with fluorescence detection. Ascorbic acid will be determined spectrophotometric reaction with dinitrophenylhydrazine. Concentrations of a- and g-tocopherol in serum will be determined by reverse-phase high performance liquid chromatography (HPLC) with fluorescence detection according to the modified method of Catignani and Biery. The activity of SOD will be analyzed by the reaction of blocking nitrotethrazolium blue reduction and nitrophormasane formation. Catalase activity measurement is based on the ability of H2O2 to produce with ammonium molybdate the color complex detected spectrophotometrically. The activity of gluthathione peroxidase will be monitored by oxidation velocity of gluthathione by Ellman reagent. Serum glucose will be analysed using the glucose-oxidase method Beckman Analyzer (Beckman Instruments Inc., Fullerton, CA, USA), IRI will be determined by radioimmunoassay using an IMMUNOTECH Insulin IRMA kit (IMMUNOTECH as, Prague, Czech Republic), C-peptide using an IMMUNOTECH C-Peptide IRMA kit (IMMUNOTECH as, Prague, Czech Republic) and glycated hemoglobin will be measured by a Bio-Rad Haemoglobin A1c Column Test (Bio-Rad Laboratories GmbH, Munich, Germany).

Fatty acid pattern in serum phospholipids will be measured after lipids extraction according to Folch and separation of lipid fractions by thin-layer chromatography. The methylesters of fatty acids will be separated by gas chromatography.

Eventual effects of different meal frequency regimens will be evaluated due to chosen gene polymorphisms.

Statistic analysis: Will be done with the use of ANOVA tests, pair and unpair t- tests and other statistic methods using standard statistic programs. Estimate of the number of subjects to be recruited was done using power analysis of repeated measurements via statistic software PASS 2005 (Number Cruncher Statistical Systems, Kaysville, UT, USA). Factors included in this model are interindividual factors (control vs. experimental group), intraindividual factors (individual time stadium in the study) and interaction between factors (divergence degree between time profiles in control and experimental group).

Study group:

50 patients with T2D

Inclusion criteria:

1. Type 2 diabetes mellitus for at least one year
2. Treatment of T2D: diet or oral antidiabetic agents (stable drug therapy at least 3 month before the trial
3. The presence of metabolic syndrome – any three of the following symptoms:
   1. Abdominal obesity – waist circumf. in men> 102 cm, in women > 88 cm
   2. Diagnosis and treatment of type 2 diabetes or raised fasting plasma glucose level (FPG> 5,6 mmol/l)
   3. Raised blood pressure (BP): systolic BP > 130 mm Hg or diastolic BP >85 mm Hg, or treatment of previously diagnosed hypertension
   4. Reduced HDL cholesterol in men < 1 mmol/l, in women < 1,3 mmol/l (or treatment)
   5. Raised triglycerides > 1,7 mmol/l (or treatment)
4. HbA1c (according to IFCC) ≥4.2 a ≤10.5%
5. Men and women aged 30-65 years
6. Body Mass Index (kg/m^2^) in the range of 27 – 50
7. The signed informed consent
8. Readiness to change dietary habits

Exclusion criteria:

1. Type 1 diabetes mellitus
2. Unstable drug therapy at least 3 month before the trial
3. Treatment with Byetta or Victosa
4. Pregnancy (positive β-HCG test), breast feeding or trying to become pregnant
5. Presence of pacemaker or other metal implant in the body (MR)
6. Alcoholism or drug use
7. Significant weight loss (more than 5% of body weight) in previous 3 months before the screening
8. Presence of other medical condition, which occurs during physical examination, laboratory tests, ECG, including pulmonary, neurological or inflammatory disease, which would be considered by the examiner to distort the consistency of data

Flowchart:

Visit 1: Screening → Randomization → Visit 2: Hyperinsulinemic isoglycemic clamp (HIC) with indirect calorimetry → Visit 3: Meal test, DXA, MRS of the liver→ Visit 4 (Oxidative stress markers assessment)

Time schedule

2010-2011 - recruiting and screening of individuals with type 2 diabetes, investigations according to study protocol (meal tests, clamps, indirect calorimetry, MRI, blood samples taking).

2012 - continuing of the study according to study protocol, initiation of blood samples analysis and MRI pictures evaluation, preliminary statistic analysis

2013 - finishing of the investigation program, finishing of laboratory analysis and statistic analysis, MRI evaluation, final statistic analysis and preparation of scientific publications.

**Appendix 1. Information for patients and informed consent**

The Effect of Meal Frequency in a Reduced-energy Regimen on Insulin Resistance, Insulin Secretion, Hepatic Fat Content, Gastrointestinal and Appetite Hormones in Subjects with Type 2 Diabetes

Aim of the study is to test whether it is healthier for patients with type 2 diabetes to eat small frequent meals or less-frequent larger meals. We want to help you and make the program as convenient for you as possible. All meals during the study will be provided for you. The study will take six months: three months of eating six meals a day, and three months of eating two meals a day (changing both regimens in a random order). Total daily caloric intake during both regimens will be the same. Participation on our study means absolving of following investigations at start, and at the end of every 3 months (week 0, 12, and 24):

Screening is a short visit where we find out if you meet our inclusion criteria. We will measure your height and weight and take blood samples for common laboratory tests.

Meal test which will take about 3,5 hours - we will take a blood sample, then give you breakfast and take other blood samples after 30, 60, 120 and 180 minutes after the meal. The physician will also examine you and answer your questions.

Magnetic resonance imaging of the liver is a noninvasive investigation with no radiation, no pain which seems similar to X-ray. It will take about half an hour.

Hyperinsulinemic isoglycemic clamp with indirect calorimetry will take about 4 hours - it will start with indirect calorimetry where you will breathe into encapsulated space for 45 minutes. After that you will get two plastic cannulas and you will be applied glucose and insulin infusion for 3 hours with blood glucose measurements every 5 minutes. Blood glucose will be kept in normal range.

DXA (Dual energy X-ray absorptiometry) scan to measure total body composition and fat content uses X-rays. However, the [radiation](http://en.wikipedia.org/wiki/Radiation) dose is approximately 1/10th that of a standard chest X-ray. It will take about ten minutes.

During a one-day-stay in the hospital at the end of each regimen, blood samples will be taken every three hours during the whole day to measure insulin secretion and glycemia. Urine will be collected during the whole day to measure microalbuminuria and C-peptide waste.

Eventual effects of different meal frequency regimens will be evaluated due to chosen gene polymorphisms.

All the above explained investigations do not make up any risk (not counting low blood sugar levels during the first 2 hours after clamp if you do not follow instructions given by health professionals). There is no testing of new drugs. All of the above are functional investigations that will not result in treatment changes. You can withdraw from our study whenever you want to without giving reason for doing so and without consequences for your further treatment. All documents related to the study will be anonymous and will be archived.

I have read and understood all the above information regarding the study and I have had the opportunity to discuss it with Dr...............

I agree to participate in this study.

Name ______________________

Signature ______________________
