# Supplementary material for: The effect of meal frequency in a reduced-energy regimen on the gastrointestinal and appetite hormones in patients with type 2 diabetes: A randomised crossover study
Source: PLoS One. 2017 Apr 3;12(4):e0174820. doi: 10.1371/journal.pone.0174820 (PMC5378398; doi:10.1371/journal.pone.0174820)
Supplement: S3 File — (DOC) [file pone.0174820.s003.doc]

**Vliv frekvence jídel na inzulínovou rezistenci, sekreci inzulínu, jaterní steatosu a sekreci gastrointestinálních a chuťových hormonů u pacientů s diabetem 2. typu**

Úvod

Inzulinová rezistence (IR) se velmi pravděpodobně uplatňuje při rozvoji diabetu 2. typu a širokého spektra klinických biochemických a humorálních odchylek, která shrnujeme pod název metabolický syndrom. Snahy o snížení IR jsou pilířem léčby nejen diabetu 2. typu, ale i celého syndromu. Mezi základní léčebná opatření u jedinců s nadváhou a obezitou patří nepochybně režimová opatření, t.j redukční dieta a zvýšení fyzické aktivity. V dietě však kromě omezení kalorického příjmu může hrát roli i frekvence jídel. V současné době se diskutuje o tom, zda je zdravější jíst častěji menší jídla, nebo méně často větší jídla.

Velikost a frekvence jídel jsou základními aspekty výživy a mohou mít značný vliv na zdraví a dlouhověkost laboratorních zvířat. U lidí je nadměrný příjem energie spojen se zvýšeným výskytem kardiovaskulárních chorob, diabetu a některých druhů rakoviny a je hlavní příčinou invalidity a úmrtí v industrializovaných zemích. Na druhou stranu vliv frekvence jídel na lidské zdraví a dlouhověkost není jasný. Jak restrikce kalorií, tak snížená frekvence jídel či přechodné hladovění může u hlodavců zabránit rozvoji různých nemocí a prodloužit délku života pomocí mechanismů, které zahrnují snížené poškození pomocí oxidačního stresu a zvýšenou odolnost ke stresu.

Zdá se, že mnohé pozitivní účinky kalorické restrikce (KR) a půstu (hladovění) jsou zprostředkovány nervovým systémem. Například přechodný půst způsobí na zvířecích modelech neurodegenerativních onemocnění zvýšenou produkci BDNF (brain-derived neurotrophic factor), který zvyšuje odolnost neuronů v mozku vůči dysfunkci a degeneraci; kaskáda BDNF může také zprostředkovávat pozitivní účinky přechodného půstu na regulaci glykémie a kardiovaskulární funkce (Mattson 2005b).

Pokusy na zvířatech velmi podporují antidiabetický vliv KR a diet s přechodným půstem. Režimy s přechodným půstem, jako je EODF (every-other-day fasting, každý druhý den půst, nejčastěji používaný protokol ve studiích se zvířaty), mohou prodloužit délku života u krys i myší (Carlson and Hoelzel 1946, Goodrick et al. 1982). KR i EODF snižují u hlodavců plazmatické hladiny glukózy a inzulínu a zlepšují glukózovou toleranci (Anson et al. 2003, Wang et al. 1997). V současnosti se velmi debatuje, zda je zdravější jíst častěji menší jídla, nebo méně často větší jídla (Mattson 2005a). Ačkoli se uvádí, že pacienti s diabetem jsou lépe schopní " mít pod kontrolou" glykémie, když jedí pravidelně malá jídla, přechodná dieta s velmi nízkým obsahem kalorií vedla u pacientů s diabetem 2. typu efektivněji k váhovému úbytku a kontrole glykémií než mírná KR (Williams et al. 1998). Navíc dva různé režimy využívající půst (EODF a půst dva dny v týdnu) snížil incidenci diabetu u krys Brattleboro (Pedersen et al. 1999). Zdá se, že mechanismem zodpovědným za antidiabetický účinek KR a přechodného půstu je zvýšená inzulínová senzitivita (Granberry and Fonseca 1999, Anson, Jones, and de Cabod 2005).

Epidemiologická data naznačující pozitivní vliv zvýšené frekvence jídel na riziko kardiovaskulárních chorob, hladinu lipidů a lipoproteinů u zdravých jedinců mohou být zpochybněna vzhledem k chybám spojeným s metodou sběru dat (Mann 1997). Studie zabývající se frekvencí jídel u pacientů s diabetem 2. typu jsou velmi omezené jak trváním sledování, tak i počtem sledovaných pacientů. Dlouhodobější studie, která porovnávala účinek tří a devíti jídel denně (každá perioda trvala čtyři týdny) u 13 pacientů s diabetem 2. typu, nepotvrdila pozitivní účinky zvýšené frekvence jídel u pacientů s diabetem 2. typu (Arnold, Mann, and Ball 1997). Než budeme moci dávat konkrétní doporučení ohledně frekvence jídel, je zapotřebí dlouhodobějších studií s větším počtem sledovaných jedinců.

Cíle projektu

Cílem naší studie je

1) zjistit vliv frekvence jídel (šesti vs. dvou jídel denně se stejnou kalorickou restrikcí -500 kcal/den) na inzulínovu rezistenci, sekreci inzulínu a jaterní steatosu.

2) charakterizovat některé mechanismy různých frekvencí jídla (množství viscerálního tuku, obsah tuku v játrech, sérové koncentrace adipokinů, střevních hormonů, markerů oxidačního stresu).

3) zjistit schopnost účastníků studie zachovat stejnou kalorickou restrikci při obou režimech, když budou důkladně edukováni a budou si připravovat jídla sami, ve srovnání s těmi, kterým budou všechna jídla zajištěna.

Půjde o randomizovanou crossover studii, kde 50 pacientů s diabetem 2. typu vystřídá v náhodném pořadí dva režimy: šest vs. dvě jídla denně. Každá perioda bude trvat tři měsíce.

Metabolismus glukózy a lipidů a jejich regulace budou důkladně vyšetřeny na začátku a na konci každé tříměsíční periody (meal test, hyperinzulínový izoglykemický clamp, nepřímá kalorimetrie, MRI jater, DXA, stanovení sérových koncentrací vybraných adipokinů, střevních hormonů a markerů oxidačního stresu).

Hypotéza

Naše hypotéza je, že nízké plazmatické koncentrace inzulínu (jako výsledek period půstu) sníží inzulínovu rezistenci a obsah tuku v játrech. Naopak častá jídla (a následné vyšší plazmatické koncentrace inzulínu) povedou k vyššímu hromadění tuku v játrech a k vyšší inzulínové rezistenci. Nižší frekvence jídel bude spojena s vyšší koncentrací střevních hormonů, které zvyšují sytost po jídle - glucagon-like peptide -1 (GLP-1), peptide YY (PYY) and pancreatic polypeptide (PP) and vyššímu útlumu ghrelinu po jídle. Méně porcí během dne bude spojeno s nižší hladino markerů oxidačního stresu.

Další hypotézou je, že účastníci studie budou mít (přes důkladnou edukaci) vyšší kalorický příjem při vyšší frekvenci jídel, když si budou jídla připravovat sami, ve srovnání s těmi, kterým budou všechna jídla zajištěna.

Klíčová slova

Inzulínová rezistence, frekvence jídel, diabetes mellitus 2. typu, nealkoholická steatohepatitis, adipokiny, střevní hormony, oxidační stres

Metodika a způsob získávání dat

Design studie: Vliv frekvence jídel na IR, sekreci inzulínu a jaterní steatosu bude sledován v rámci randomizované crossover studie, do níž bude zařazeno 50 nemocných s diabetem 2. typu, kteří budou náhodně měnit následující dva jídelní režimy: šest jídel a dvě jídla denně. U obou režimů bude stejná kalorická restrikce (-500 kcal/den). Každá perioda daného jídelního režimu bude trvat tři měsíce. Pro polovinu účastníků budou všechna jídla během celé studie zajištěna. Druhá poloviny účastníků bude důkladně edukována ohledně zachování kalorického příjmu při obou režimech a budou si jídla připravovat sami.

Soubor. Zařazeno bude 50 nemocných s diabetem 2. typu, léčených dietou nebo perorálními antidiabetiky, s trváním diabetu nejméně jeden rok, ženy i muži, ve věku 30-65 let, s BMI 27-50 kg/m2. Vyšetřovaným budou vysvětleny cíle, metody a rizika studie a podepíší informovaný souhlas (Příloha 1).

Jídelní režimy: Při režimu šesti jídel za den budou účastníci požádáni, aby rozdělili svůj celkový kalorický příjem do šesti jídel a aby jedli každé dvě až tři hodiny. Při režimu dvou jídel za den rozdělí účastníci studie svůj celkový kalorický příjem do dvou jídel: první jídlo budou jíst mezi 6. a 10. hodinou dopoledne a druhé jídlo mezi 12. a 16. hodinou.

Pohybový režim: Účastníci budou požádáni, aby neměnili své pohybové návyky během studie. Fyzická aktivita bude sledována za použití krokoměrů a standardizovaných dotazníků: IPAQ (International Physical Activity Questionnaire) a Baeckeho dotazníku habituální pohybové aktivity.

Vyšetření

Na začátku studie (týden 0) a po každých 3 měsících intervence (týden 12 a 24) budou provedena následující vyšetření (u každého jedince celkem 3x):

1. Běžná antropometrická vyšetření (hmotnost, BMI, obvod pasu a boků) a odběry krve pro běžná laboratorní vyšetření, zhodnocení metabolismu glukózy a lipidů, stanovení koncentrací vybraných adipokinů, střevních hormonů, markerů oxidačního stresu, vitaminů, složení mastných kyselin ve fosfolipidech séra (viz. analytické metody).

2. Hyperinzulinový (1 mU/kg/min) izoglykemický clamp (HIC) v trvání 3 hodiny s nepřímou kalorimetrií. Metoda umožňuje přesnou kvantifikaci inzulínové rezistence a utilizace energetických substrátů.

3. Meal test pro stanovení glukózové tolerance a sekrece inzulinu po standardní snídani /bageta Crocodille Sýrový mlsoun - 180g, energie 452,8 Kcal/1895,7 kJ, složení: sacharidy 49,2 g (44,55%), proteiny 18,5 g (16,74%), lipidy 18,8 g (38,7%), z toho saturované 6,8 g, monoenové 6,0 g, polyenové 5,0 g/. Odběry ke stanovení glykémie, C peptide, imunoreaktivního inzulinu (IRI) se provádějí v 0, 30, 60, 120 a 180 minutě po snídani.

4. DXA (Dual energy X-ray absorptiometry) ke stanovení složení těla a obsahu tuku.

5. MRI (magnetic resonance imaging) jater ke kvantifikaci obsahu tuku v játrech.

6. Odběry oxidační stres

Analytické metody

Hladiny vybraných adipokinů v séru (resistinu, celkového adiponectinu, HMW adiponectinu, TNF a leptinu) případně dalších cytokinů a proteinů (FABP) dle stavu poznání budou hodnoceny metodou ELISA (Linco, USA). hsCRP bude měřen pomocí imunoturbidimetrie. Markery oxidačního stresu: Parametry lipoperoxidace budou zhodnoceny podle hladin konjugovaných dienů a koncentrací látek reagujících s kyselinou thiobarbiturovou metodou (popsanou Beugem a Austeem) s fluorometrickou detekcí. Koncentrace oxidovaných forem glutationu budou stanoveny kolorimetrickými metodami s použitím Ellmanova reagens, koncentrace redukovaného glutationu meřením snížení koncentrace NADPH. Metodou HPLC budou stanoveny koncentrace vitaminů a zhodnocena bude aktivita katalázy a superoxiddismutázy. Glykémie ve venózní krvi bude vyšetřena glukózooxidázovou metodou analyzátorem Beckman Analyzer (Beckman Instruments Inc., Fullerton, CA, USA), hladiny imunoreaktivního inzulinu radioimunologicky pomocí IMMUNOTECH Insulin IRMA kitu (IMMUNOTECH as, Praha, ČR), C-peptide pomocí IMMUNOTECH C-Peptid IRMA kitu (IMMUNOTECH as,Praha, ČR) a glykovaný hemoglobin pomocí Bio-Rad Haemoglobin A1c Column Testu (Bio-Rad Laboratories GmbH, Munchen, Germany). Po extrakci lipidů ze séra podle Folcha a rozdělení jednotlivých frakcí chromatografií na tenké vrstvě bude provedena analýza metylesterů jednotlivých mastných kyselin ve fosfolipidech séra metodou plynové chromatografie.

Případný vliv různých jídelních režimů bude zhodnocen vzhledem k vybraným genovým polymorfismům.

Statistické zpracování. Bude provedeno pomocí testů ANOVA, párových a nepárových t testů a dalších statistických metod za použití standardních statistických programů. Kalkulace počtu zařazených osob byla provedena na základě silové analýzy opakovaných měření pomocí statistického softwaru PASS 2005 (Number Cruncher Statistical Systems, Kaysville, UT, USA). Faktory zahrnuté v tomto modelu jsou faktory interindividuální (kontrolní vs. experimentální skupina), intraindividuální (individuální časová stadia ve studii) a interakce mezi faktory (míra divergence mezi časovými profily v kontrolní a experimentální skupině).

**Studijní skupina:** 50 diabetiků 2. typu
    Kritéria pro zařazení:
    1. Pacient má cukrovku 2. typu déle než 1 rok.
    2. Léčba cukrovky: dieta či PAD  stabilně v posledních 3 měsících

    3. Pacient splňuje diagnostická kritéria metabolického syndromu – tzn. nejméně 3 z těchto rizik. faktorů:

1. abdominální obezita – obvod pasu muži > 102 cm, ženy > 88 cm
2. krevní tlak léčený nebo >130/85 mm Hg
3. cukrovka nebo porucha glukózové tolerance nebo glykémie nalačno > 5,6 mmol/l
4. HDL cholesterol – léčba nebo muži < 1 mmol/l, ženy < 1,3 mmol/l
5. Triglyceridy – léčba nebo > 1,7 mmol/l

4. HbA1c ≥4.2 a ≤10.5% dle IFCC

    5. Ženy i muži ve věku 30-70 let

    6. Body Mass Index (kg/m^2^) mezi 27 a 50

    7. Informovaný souhlas: podepsaný, s udáním data

    8. Ochota změnit dietní návyky a dodržovat předepsanou dietu

    Kritéria pro vyřazení:

    1. Současné kouření, alkoholismus nebo užívání drog

    2. Těhoteství, kojení

    3. Nestabilní medikace na cukrovku v posledních 3 měsících, nestabilní TK či nestabilní léčba dyslipidemie v posledních 3 měsících.

    4. Diagnóza diabetu 1. typu

    5. Významný váhový přírůstek či úbytek (> 5% celkové tělesné hmotnosti) během posledních 3 měsíců před screeningem

    6. Kardiostimulátor nebo kovový implantát v těle

7. Aplikace inzulínu, Byetty nebo Victózy

**Plán studie:**

Vyšetření v 0, 3 a 6 měsících

**Návštěva 1: Screening → Randomizované zařazení do studie → Návštěva 2: hyperinzulinemický euglykemický clamp a nepřímá kalorimetrie → Návštěva 3: Meal test, DXA, MRS jater → Návštěva 4 (Oxidační stres)**

**Návštěva 1: Screening**

1. Vyplnit **Screening (Příloha 2)**. Probrat všechna kritéria pro zařazení a pro vyřazení (bod po bodu)

2. Probrat s pacientem a podepsat **informovaný souhlas (Příloha 1)**

3. **Zvážit, změřit, spočítat BMI**

4. Změřit **TK**

5. **Odběry** - Na, K, Cl, urea, kreatinin, bilirubin, ALT, AST, GMT, HbA1c, HIV, HbsAg, chol, TG, HDL chol, LDLchol, anti GAD protilátky

6. Objednat pacienta na Návštěvu 2 (clamp + nepřímá kalorimetrie), Návštěvu 3 (meal test, DXA, MRS jater) a na Návštěvu 4 (odběry oxidační stres), premenopauzální ženy objednat na návštěvy 2+3 1. týden po menstruaci, poučit pacienta o lačnění na obě návštěvy, poučit o sběru moče na mikroalbuminurii, na Návštěvu 2, poučit o vysazení metforminu večer  a ráno před clampem.

**Randomizované zařazení do studie**

**Návštěva 2: hyperinzulinemický euglykemický clamp a nepřímá kalorimetrie** (Příloha 6)

**Protokol viz základní schéma**

**Postup.**

Vyšetřovaný se dostaví do LKP (cca 7 hod) – nalačno, přinese s sebou 24 hodinovou porci moče (4 vzorky + množství moče ..zapsat v minutách a ml)

**Zavedeme 2 kanyly k infuzím a odběrům**

**Provedeme nepřímou kalorimetrii** (celkem v trvání asi 1hod a 30 min)

kalibrace asi 30-45 min

vlastní měření 45 min

**Hyperinzulinový izoglykemický clamp (1 mU/kg/min; 5,5 - 8 mmol/l, 3 hodiny)**

Po odběrech krve a změření TK zahájíme infuzi inzulinu rychlostí 2 (10 min) a po t é 1 mU/kg/min.

Infuze glukózy bude zahájena až po poklesu glykémie na hodnoty 5,5 – 8 mmol/l (cílová glykémie bude vypočítána jako lačná glykémie – 1,5 až 4 mmol/l)

**TK:** 0‘ – TK 3x, první hodnotu nezapočítávat, vypočítat průměr z druhých 2 měření

180‘ - TK 3x, první hodnotu nezapočítávat, vypočítat průměr z druhých 2 měření

**Sběry moče** : 24 h (06-06) **množství ......... čas (min)...........**

Poslat ke stanovení : MAU, GF, odpady urey

**Odběry:**

urea, kreat - 0 min

genetika - 0 min

cholesterol, HDL-cholesterol, LDL-cholesterol, triglyceridy, Apo A1, Apo B – 0´ (Piťha) 0

IRI - 0, 150, 165, 180

10 kepů á 0,2 ml sera do krabiček na následující stanovení – 0 min.

MK ve fosfolipidech sera (1ml séra) - 0 min

**Návštěva 3: Meal test, DXA, MRS jater**

**1.** Vyplnit **Přílohu č. 4**

**2. Meal test**  test pro stanovení glukózové tolerance a sekrece inzulinu po standardní snídani

**Provedení -** na lačno (po 8-12 hod lačnění) v LKP, po odběrech podat standardní snídani a provést další odběry dle rozpisu

**Standardní snídaně** – bageta Crocodille Sýrový mlsoun – 180g, energie 452,8 Kcal/1895,7 kJ, složení: sacharidy 49,2 g (44,55%), proteiny 18,5 g (16,74%), lipidy 18,8 g (38,7%), z toho saturované 6,8 g, monoenové 6,0 g, polyenové 5,0 g.

**Odběry.**

Glykémie – 0, 30, 60, 120 a 180 min.

IRI – 0, 30, 60, 120 a 180 min.

C-peptid– 0, 30, 60, 120 a 180 min.

TG + NEMK - 0, 30, 60, 120 a 180 min.

GI peptidy (ghrelin, leptin, GIP, GLP-1, amylin, PP, PYY- 0, 30, 60, 120 a 180 min. do spec. zkumavek s činidly – stočit, uložit do krabiček á 2x0,3 ml, do -80 st. C

Zálož. sérum - 0, 30, 60, 120 a 180 min. – stočit, uložit do krabiček á 2x0,3 ml, do -80 st. C

3. Vstupní **lékařské vyšetření**

4. Pacient vyplní **dotazníky** (Příloha 9-14)

**Návštěva 4 (Oxidační stres)**

1. Zvážit, změřit pas a boky
2. Odběry
3. **markery oxidačního stresu** – SOD, CAT, GPx, GR, GST, αE, γE, AA, GSH, TBARS, CD – **1 velká fialová zkumavka + 1 malá červená zkumavka s gelem** – ihned po odběru do lednice, nestáčet, do 1h donést na Z6 – Mgr. Malínská
4. **HbA1c** – šedá zkumavka
5. **Fe, transferin, ferritin, TIBC** – **1 malá červená zkumavka**
6. **Homocystein** – **4 ml plné krve do** **zkumavky s EDTA (velká fialová)**, **uložit hned do lednice či ledové tříště**, poté do 30 min. stočit, 0.5 ml plazmy dát do kepu, popsat a uložit v -80ºC, po dohodě konkrétního termínu převézt všechny vzorky najednou Dr. Tvrzické, VFN Karlovo nám., tel. 224962500, eva.tvrzicka@vfn.cz

**Příloha 1. Informace pro nemocné a informovaný souhlas**

**Vliv frekvence jídel na inzulínovou rezistenci, sekreci inzulínu a jaterní steatosu u nemocných s diabetem 2. typu**

Cílem naší studie je zjistit, zda je pro pacienty s diabetem 2. typu zdravější jíst častěji malá jídla, či méně často větší jídla. Chceme Vám maximálně pomoci v dodržování jednotlivých jídelních režimů. Během studie budete mít možnost odebírat si připravená jídla. Studie bude trvat 6 měsíců: tři měsíce budete jíst šest menších jídel denně, další tři měsíce dvě větší jídla denně (oba režimy se vystřídají v náhodném pořadí). Celkový kalorický příjem za den bude stejný. Účast na této studii znamená absolvovat následující vyšetření na začátku a po každých 3 měsících daného jídelního režimu (týden 0, 12 a 24):

Screeningové vyšetření, které rozhodne o tom, zda splňujete vstupní kritéria pro zařazení do studie. Patří sem zvážení, změření a základní odběry krve, probrání vstupních kritérií pro zařazení do studie.

Vstupní vyšetření, které bude trvat asi 3,5h, spočívá v dalším odběru krve, dostanete malou snídani a po 30, 60, 120 a 180 min. Vám odebereme další vzorky krve. Při této návštěvě Vás také vyšetří lékař a projde s Vámi případné nejasnosti, které máte ohledně studie.

Na pracovišti magnetické rezonance IKEM bude provedeno MRI jater ke stanovení obsahu tuku v játrech - jde o zcela nebolestivé vyšetření bez radiační zátěže, které vypadá jako rentgen. Vyšetření bude trvat asi půl hodiny.

Hyperinzulínový izoglykemický clamp doplněný nepřímou kalorimetrií bude celkem trvat asi 3h - prakticky jde o 3 hodiny trvající infuzi glukózy a inzulinu v dávce asi 3 j/hodinu za průběžných kontrol hladin krevního cukru. Vyšetření probíhá vleže. Vyšetřovaný má zavedenou plastovou kanylku v předloketní žíle, kam kapou infuze, a druhou umístěnou v žíle na hřbetě ruky, kde je každých 10-15 min odebírána 1 kapka krve ke stanovení krevního cukru. Krevní cukr je udržován na hladině 5 mmol/l. 45 minut před zahájením clampu bude provedena nepřímá kalorimetrie, při níž budete dýchat do uzavřeného prostoru.

DXA (Dual energy X-ray absorptiometry) ke stanovení složení těla a obsahu tuku. Při tomto vyšetření se využívá rentgenových paprsků, radiační zátěž dosahuje pouze 1/10 zátěže při standardním rentgenovém snímku hrudníku. Vyšetření trvá cca 10 min.

Sekrece inzulínu a glykémie budou měřeny během jednoho dne v nemocnici na konci každé režimové periody. Vzorky krve budou odebírány každé tři hodiny po celý den. Po celý den bude také sbírána moč ke stanovení mikroalbuminurie a odpadů C-peptidu.

Případný vliv různých jídelních režimů bude zhodnocen vzhledem k vybraným genovým polymorfismům.

Výše uvedená vyšetření nejsou spojena s žádným rizikem (vyjma nízké hladiny krevního cukru - hypoglykémie do 2 hodin po ukončení hyperinzulinového clampu, pokud nedodržíte pokyny zdravotního personálu). Nejedná se o žádné zkoušení nového léku či přípravku. Jde pouze o funkční vyšetření, které nebude mít bezprostřední dopad na vaši léčbu. Kdykoli během sledování můžete bez udání důvodů ze studie odstoupit, aniž by ukončení Vaší účasti mělo dopad na případnou léčebnou péči. A všechny dokumenty související se studií budou anonymní a budou uchovávány.

Přečetl jsem a porozuměl výše uvedené informaci o studii a měl jsem možnost o ní hovořit s Dr.................................

Potvrzuji, že souhlasím s účastí v této studii.

Jméno ______________________

Podpis ______________________
